# Relevance of sociodemographic characteristics on patients with bipolar disorder and substance use disorder

**DOI:** 10.1192/j.eurpsy.2024.631

**Published:** 2024-08-27

**Authors:** I. A. Silva, C. Silva, I. Faria, V. S. Melo

**Affiliations:** ^1^Unidade Local de Saúde do Norte Alentejano, Portalegre; ^2^Centro Hospitalar e Universitário de Coimbra, Coimbra; ^3^Centro Hospitalar do Médio Tejo, Tomar, Portugal

## Abstract

**Introduction:**

Substance use disorder is a common comorbidity with bipolar disorder, having implications on its diagnosis, treatment adherence, and number of hospitalizations. Understanding the particular characteristics of this population is of the utmost importance to improve clinical outcomes.

**Objectives:**

Our aim is to analyze the sociodemographic characteristics of the patients in the inpatient unit of a tertiary hospital and to reflect on its impact on treatment. Our study looks over a 3-year period, and all patients analyzed have a dual diagnosis of both bipolar disorder and substance use disorder.

**Methods:**

We collected, retrospectively, data from the hospital platform and analyzed it on SPSS Statistics 26, along with a literature review.

**Results:**

In the analyzed period of 3 years, there were 2384 hospitalizations in the Coimbra’s University Hospital psychiatric ward, and 88 hospitalizations were coded with a dual diagnosis of bipolar disorder and substance use disorder.

Regarding gender distribution, 41% of the patients were female and 49% of the patients were male, with a mean age of 47 years.

There were 12 patients who were re-hospitalized once (7 of them were men) and 6 who were re-hospitalized twice (4 of them were men) during the analyzed period.

At the time of hospitalization, 60.5% of male patients were single, 21.1% were divorced, and only 15.8% were married, while female patients were mainly married (35.7%) and only 28.6% were single.

Female patients had more frequent support from social and community institutions (17.9% vs 5.3% in men) while 2.6% of men had no support from family or institutions.

Regarding education, more men accomplished high school education (21.1% vs 17.9% in women) and university education (18.4% vs 14.3%). In our sample, there were 3.6% of women who were illiterate.

During their lifetime, female patients were hospitalized around 5 times and men around 3.7 times, despite the fact that the mean age of female patients on their first hospitalization was 36 years, and in male patients, it was 34 years.

Treatment adherence is more significant in female patients (70.3% vs 69.2% in men), even though women maintain active substance abuse more frequently (42.9 vs 39.5%).

**Conclusions:**

Male and female patients have different backgrounds and different support either in spouses, family, or social institutions. These nuances may play an important role in the number of re-hospitalizations, treatment adherence, and maintenance of abstinence.

Taking these aspects into consideration may help improve clinical outcomes.

**Disclosure of Interest:**

None Declared

